# A randomised controlled trial assessing the use of citalopram, sertraline, fluoxetine and mirtazapine in preventing relapse in primary care patients who are taking long-term maintenance antidepressants (ANTLER: ANTidepressants to prevent reLapse in dEpRession): study protocol for a randomised controlled trial

**DOI:** 10.1186/s13063-019-3390-8

**Published:** 2019-06-03

**Authors:** Larisa Duffy, Faye Bacon, Caroline S. Clarke, Yvonne Donkor, Nick Freemantle, Simon Gilbody, Rachael Hunter, Tony Kendrick, David Kessler, Michael King, Paul Lanham, Gemma Lewis, Dee Mangin, Louise Marston, Michael Moore, Irwin Nazareth, Nicola Wiles, Glyn Lewis

**Affiliations:** 1 Division of Psychiatry, University College London, 6th Floor Maple House, 149 Tottenham Court Road, London, W1T 7NF UK; 2 Research Department of Primary Care and Population Health, University College London, UCL Medical School, Upper 3rd Floor, Royal Free Campus, Rowland Hill Street, London, NW3 2PF UK; 3 Priment Clinical Trials Unit, University College London, UCL Medical School, Upper 3rd Floor, Royal Free Campus, Rowland Hill Street, London, NW3 2PF UK; 4 Comprehensive Clinical Trials Unit, University College London, 90 High Holborn 2nd Floor, London, WC1V 6LJ UK; 5 Department of Health Sciences, University of York, Seebohm Rowntree Building, Heslington, York, YO10 5DD UK; 6 Primary Care & Population Sciences, University of Southampton, Aldermoor Health Centre, Southampton, SO16 5ST UK; 7 Centre for Academic Mental Health, Population Health Sciences, Bristol Medical School, University of Bristol, Oakfield House, Oakfield Grove, Bristol, BS8 2BN UK; 8 Department of Family Medicine, McMaster University, 1280 Main Street West, Hamilton, Ontario L8S 4L8 Canada; 9 Department of General Practice, University of Otago, Christchurch, PO Box 4345, Christchurch, 8140 New Zealand

**Keywords:** Depression, Primary care, Antidepressants, Sertraline, Citalopram, Fluoxetine, Mirtazapine, Selective serotonin reuptake inhibitors

## Abstract

**Background:**

Antidepressants are used both for treating acute episodes and for prophylaxis to prevent future episodes of depression, also called maintenance treatment. This article describes the protocol for a randomised controlled trial (ANTLER: ANTidepressants to prevent reLapse in dEpRession) to investigate the clinical effectiveness and cost-effectiveness in UK primary care of continuing on long-term maintenance antidepressants compared with a placebo in preventing relapse of depression in those who have taken antidepressants for more than 9 months and who are currently well enough to consider stopping maintenance treatment.

**Methods/design:**

The ANTLER trial is an individually randomised, double-blind, placebo-controlled trial in which participants are randomised to remain on active medication or to take an identical placebo after a tapering period of 2 months. Eligible participants are those who: are between the ages of 18 and 74 years; have had at least two episodes of depression; and have been taking antidepressants for 9 months or more and are currently taking citalopram 20 mg, sertraline 100 mg, fluoxetine 20 mg or mirtazapine 30 mg but are well enough to consider stopping their medication. The participants will be followed up at 6, 12, 26, 39 and 52 weeks.

The primary outcome will be the time in weeks to the beginning of the first episode of depression after randomisation. This will be measured using a retrospective version of the Clinical Interview Schedule—Revised administered at 12, 26, 39 and 52 weeks.

Secondary outcomes will include depressive and anxiety symptoms, adverse effects, withdrawal symptoms, emotional processing tasks, quality of life and the resources and costs used. We will also perform a cost-effectiveness analysis based on results of the trial.

**Discussion:**

The ANTLER trial findings will inform primary care prescribing practice by providing a valid and generalisable estimate of the clinical effectiveness and cost-effectiveness of long-term maintenance treatment with antidepressants in UK primary care.

**Trial registration:**

Controlled Trials ISRCTN Registry, ISRCTN15969819. Registered on 21 September 2015.

**Electronic supplementary material:**

The online version of this article (10.1186/s13063-019-3390-8) contains supplementary material, which is available to authorized users.

## Background

Depression is a major health problem that is not only debilitating to the individual but also to society, being the lead cause of disability worldwide [1]. Globally, more than 300 million people live with depression. Antidepressants are often a first-line treatment for depressive symptoms and are also used for maintenance treatment; that is, to prevent relapse once an individual has recovered. It has been estimated that, between 1993 and 2005, 90% of antidepressant prescriptions [2] in the UK were used for maintenance. A more recent UK study [3] has also demonstrated a steady increase between 2001 and 2012 in the duration of long-term treatment.

The number of prescriptions for antidepressants has risen dramatically in recent years; increasing by around 7% per annum in the UK. Furthermore, antidepressant prescribing has shown a greater increase than drugs for any other therapeutic area, with over 65 million prescriptions being issued in England in 2016, at a cost of £266.6 million to the NHS [4, 5] Similar increases in antidepressant prescribing have been observed in other high-income countries [6].

Moreover, there are other considerations in addition to cost. Prolonged antidepressant treatment has been associated with common side effects such as weight gain, sleep disturbance and sexual dysfunction. There are also reports of an association between antidepressants and severe adverse outcomes in older people such as stroke and transient ischaemic attack, although there is no evidence to say that the associations are causal [7].

The National Institute for Health and Care Excellence (NICE) in England recommends that antidepressant maintenance treatments should continue to be used for 2 years for those at risk of relapse [8]. However, they also recognise the uncertainty about the benefit of long-term maintenance treatment and recommend further research into its psychological and pharmacological effects.

The impact on relapse rates of continuing maintenance treatment in the first few months after remission has been achieved with antidepressant treatment has been extensively studied [9–11]. However, the amount of evidence for a treatment period longer than 36 weeks is small. In the existing reviews, there were only three studies [12–14] that have treated patients for more than 32 weeks. All three had methodological and statistical limitations due to either small sample size (e.g. Cook et al. [12], *N* = 15; Bialos et al. [13], *N* = 17; and Kupfer et al. [14], *N* = 20) or sample characteristics (e.g. Cook et al. [12] sample compromised elderly males). A further weakness in the reviewed studies was that most were funded by the pharmaceutical industry. The pharmaceutical companies can be imaginative in ways of manipulating their research findings [15, 16] and there is evidence that they publish only half of their trials [17]. The studies were conducted in a variety of different health systems with antidepressant medication that is not currently used in the UK. Therefore, the results are difficult to generalise to the UK population.

There is some evidence that the number of previous episodes, a presence of residual depressive symptoms and female gender are associated with increased rates of relapse. However, there is no evidence that these factors are associated with the difference in relapse rates between maintenance antidepressant and placebo.

The long-term benefits of the ANTLER (ANTidepressants to prevent reLapse in dEpRession) trial may lead to improving treatment recommendations and guidance for general practitioners (GPs). As there is very limited evidence for effectiveness of maintenance treatment longer than 6 months, the ANTLER trial will refine our understanding of the costs and benefits of long-term maintenance therapy and therefore will help to inform patients and practitioners when treatment decisions regarding the duration of treatment are being discussed. If the results of the ANTLER trial demonstrate that long-term maintenance treatment proves ineffective, this will lead to benefits associated with reducing not only unnecessary treatment but also adverse effects and costs. On the other hand, if maintenance treatment proves effective, individuals who are not currently taking medication to prevent depressive relapse might benefit from antidepressant use.

The aim of the trial is to answer the following research question: ‘What is the clinical effectiveness and cost-effectiveness in UK primary care of continuing on long-term maintenance antidepressants compared with a placebo in preventing relapse of depression in those who have taken antidepressants for more than 9 months and who are now well enough to consider stopping maintenance treatment?’

The objective of the trial is to provide a valid and generalisable estimate of the clinical effectiveness and cost-effectiveness of long-term maintenance treatment with antidepressants in UK primary care.

### The choice of trial medication

The choice of medication was guided by the pragmatics of recruitment and carrying out the study. We think it is important to compare the active treatment with a placebo in a condition such as depression with well-described placebo effects. Therefore, we wanted to minimise the number of antidepressants to make the manufacture and distribution of placebo easier. There are a large number of antidepressants all of which act on the monoamine systems, especially serotonin (5-hydroxytryptamine or 5HT) and noradrenaline. The tricyclic antidepressants are 5HT and/or noradrenaline reuptake inhibitors, although they tend also to have other pharmacological actions that increase the side effect burden. The most commonly prescribed antidepressants now are the selective serotonin reuptake inhibitors (SSRIs): citalopram, escitalopram, sertraline, paroxetine and fluoxetine. Other commonly used antidepressants include venlafaxine, which has both serotonin and noradrenaline reuptake inhibitor (SNRI) properties. Mirtazapine has a slightly different mode of action and is described as a noradrenergic and specific serotonergic antidepressant (NaSSA), although the net effect of its action is to increase serotonergic and to some extent noradrenaline transmission. Use of mirtazapine is increasing rapidly and accounted for 13% of prescriptions in England for antidepressants in 2013.

Due to marked pharmacological similarities between the antidepressants it is usually assumed that they share a common mode of action and any differences in efficacy are likely to be relatively minor [18]. Meta-analyses [10, 11] of the different classes of antidepressants have found no evidence to suggest that the tricyclics, SSRIs and SNRIs differ in their effectiveness as a maintenance treatment.

We have chosen not to use paroxetine as it has a short half-life and is associated with a more marked withdrawal syndrome and might not be tolerated by some individuals when they are withdrawn after randomisation. Escitalopram is not widely used in primary care in the UK and has not been included in many primary care formularies. Venlafaxine tends to be used more by secondary care than primary care doctors, can be poorly tolerated and also has more marked withdrawal effects. Amitriptyline is often used for treatment of pain and insomnia, and much less often now as an antidepressant because it is less well tolerated than SSRIs and potentially more lethal in overdose. Moreover, amitriptyline is not recommended as a first-line antidepressant—we have omitted it here.

We will therefore recruit primary care patients who are on maintenance treatment with the SSRIs citalopram 20 mg, sertraline 100 mg and fluoxetine 20 mg. We have also included mirtazapine 30 mg given its increasing use. Together these medications currently comprise about 75% of all long-term antidepressant prescriptions in England (personal email communication with Prof. Irene Petersen) and are all licensed for treatment of depression.

## Methods/design

### Study design

The ANTLER trial is a double-blind, individually randomised, parallel group controlled trial. We will recruit individuals in primary care who are currently on one of four of the most commonly used antidepressant medications but are currently well enough to consider stopping medication. Participants will be recruited from primary care practices in four UK sites: London, Bristol, Southampton and York.

Our trial will compare continuing the antidepressant medication (citalopram 20 mg, sertraline 100 mg, fluoxetine 20 mg or mirtazapine 30 mg) with replacement of the medication with an identical placebo after a tapering period. The trial intervention will be for 52 weeks and we will follow up the participants at 6, 12, 26, 39 and 52 weeks.

### Inclusion and exclusion criteria

#### Inclusion criteria

Eligible participants will be primary care patients who are being treated for depression; have had at least two episodes of depression; are aged 18–74 years; have been taking antidepressants for 9 months or more and are currently on citalopram 20 mg, sertraline 100 mg, fluoxetine 20 mg or mirtazapine 30 mg; and are well enough to consider stopping their antidepressant medication. We have a pragmatic approach to the ‘well enough’ definition and will not expect to have an accurate timeframe of how long patients have been feeling well prior to enrolment into the trial. To be eligible, participants must also have adhered to their medication. We will use the same criteria as used in the MIR trial to define adherence using a five-item self-report measure of compliance [4]; the questions are available from the authors on request.

#### Exclusion criteria

Participants will also be excluded if they meet internationally agreed (ICD-10) criteria for a depressive illness assessed using the CIS-R. GPs will be asked to exclude patients who have bipolar disorder, psychotic illness, dementia or a terminal illness; are unable to complete self-administered questionnaires in English; have contraindications for any of the prescribed medication; are concurrently enrolled in another investigational medicinal product (IMP) trial; are women who are currently pregnant or planning pregnancy or lactating; are using monoamine oxidase inhibitors; or have allergies to placebo excipients.

### Recruitment of participants

We plan to recruit 479 participants over 2 years from approximately 200 practices across our four research centres, based in England, using two methods: record search and in-consultation recruitment.

#### Method 1: record search

GP practice staff or NHS employed Clinical Research Network (CRN) staff will carry out record searches to identify potentially eligible patients and write to these individuals so that they can consider joining the study. The mail-out procedure will involve an initial letter sent by the GP surgery to the identified patients, followed by a reminder invitation letter if there is no response. Those patients who reply positively to the invitation letter will be reviewed by their GP, who will inform the local Principal Investigator (PI) on inclusion/exclusion criteria from the patients’ medical notes. The GP could also decide that the person was unsuitable to take part in the trial on any other grounds.

#### Method 2: in consultation

GPs will introduce the trial to suitable patients at consultation and ask for their permission for release of contact details to the study team. The information will be sent by secure email or fax to the study team. A study researcher will contact the patient to confirm eligibility for the trial and arrange the baseline visit.

### Screening of potential participants

Patients who have been identified by either method of recruitment will answer a depressive symptom questionnaire (PHQ-9 [19]) and questions on adherence to medication either over the phone, by post or by email. The PHQ-9 score will be used to indicate whether the individual is likely to meet the ICD-10 criteria for depressive illness at baseline, and therefore if the patient scores 15 and above they will not be invited to the baseline assessment. Potentially eligible patients will be invited for a baseline assessment that will establish any remaining eligibility criteria. The assessment will take place in the patient’s home, at their general practice or on university premises.

### Baseline assessment

At the baseline meeting, the researcher will explain the study in detail and obtain written informed consent for the baseline assessment. The potential participants will complete the following assessments: the Clinical Interview Schedule (CIS-R) [20] to assess ICD-10 criteria for depression, past medical history questions including any physical illness contraindications and past psychiatric treatments, and sociodemographic and other background information. The participants will be asked for details of their prescribed medication and prior use of antidepressants.

Potential participants who do not have an ICD-10 primary diagnosis of depression using the CIS-R will be told that they are potentially eligible to enter the trial (pending confirmation by PI) and will be asked to provide further consent for trial participation.

All participants invited to a baseline assessment will also complete the following questionnaires: depressive symptoms (PHQ-9), anxiety symptoms GAD-7 [21], EQ-5D-5L [22] for quality-adjusted life years (QALYs), adverse effects of antidepressants (a modified Toronto Side Effects scale) [23], adherence to study medication, health-related quality of life SF-12, and withdrawal symptoms based on the DESS [24]. Potential participants will be asked to perform computerised emotional processing tasks [25–27]. Women of child-bearing age will carry out a pregnancy test.

Once the baseline assessment is complete, final eligibility status will be confirmed by the local PI.

### Randomisation procedure and unblinding

Following completion of the baseline assessment and provision of written consent, participants will be randomised using the automated randomisation service provided by Sealed Envelope (https://sealedenvelope.com). The randomisation will be minimised by the four study centres, the four medications and the severity of depressive symptoms at baseline (two categories measured using the CIS-R). The dispensing pharmacy (University Hospitals Bristol Pharmacy) will be informed of the randomised allocation and post the medication by recorded delivery to either the participant’s home or GP surgery at regular 8-week intervals. The researcher will send a letter to the participant’s GP informing them of the patient’s enrolment into the trial. Trial participants, clinicians and all members of the research team will be blinded to the trial treatment allocation. Participants will be free to withdraw from the medication at any time.

Together with the study medication, participants will be provided with a contact card so that any treating clinician can be unblinded to treatment allocation in case of a medical emergency (‘emergency unblinding’) or early unblinding to enable treatment decisions. If unblinding is required, a formal request by a clinician will be made to the trial pharmacy (through the 24-h contact number provided on the contact card) that has a list of the participants’ treatment allocations. The treating physician will manage the medical emergency as appropriate upon receipt of the treatment allocation.

The PI or delegate will record any breaking of the code and reasons for doing so on the Case Report Form (CRF)/data collection tool and in the site file. Where possible, members of the research team should remain blinded. Those participants who have not required emergency or early unblinding will be unblinded on completion of the trial (‘routine unblinding’). This information will be provided to their GP by the pharmacy; the participant will need to consult their GP and any further treatment can be discussed during that consultation. The trial team will remain blind to this information and will not provide further supplies of the trial medication once participants have been unblinded.

### Treatment of participants

At baseline, participants will be taking either citalopram 20 mg, sertraline 100 mg, fluoxetine 20 mg or mirtazapine 30 mg. They will be randomised either to remaining on their current medication or to placebo. For those in the placebo group, in the first month they will take the same medication at half the dose (citalopram 10 mg, sertraline 50 mg, or mirtazapine 15 mg). In the second month they will take half the dose and placebo on alternate days, and from the third month until the end of the study they will take the placebo. There is no 10 mg capsule for fluoxetine so those taking fluoxetine at baseline who are allocated to the placebo arm will alternate between a 20 mg tablet and a placebo tablet for 1 month. During the second month they will take placebo as fluoxetine has a long half-life.

The active medication will be encapsulated and the placebo will be an identical capsule filled with an inert excipient. All capsules will exactly match in dimensions and appearance, so that allocation concealment and blinding is maintained.

### Subsequent assessments

Follow-up assessments will be carried out at 6, 12, 26, 39 and 52 weeks after randomisation. Participants will continue to be invited to follow-up assessments unless they have withdrawn from the trial. Participants will be followed up if they have stopped taking the study medication. Follow-up assessments will take place either at the participant’s home, at the general practice or on university premises. The dates of the assessments will be recorded and the analysis plan will include measures to investigate any influence of the timing of the follow-up appointments.

#### Follow-up assessment schedule

At 6 weeks post randomisation, the participants will be asked to complete a postal questionnaire.

At 12, 26, 39 and 52 weeks after randomisation, the participants will be asked to attend an appointment with the researcher.

After 52 weeks, primary healthcare use data (prescribed medication, primary care visits) for the time period of the trial and for 6 months preceding the trial will be extracted from GP electronic health records.

**Fig. 1:**
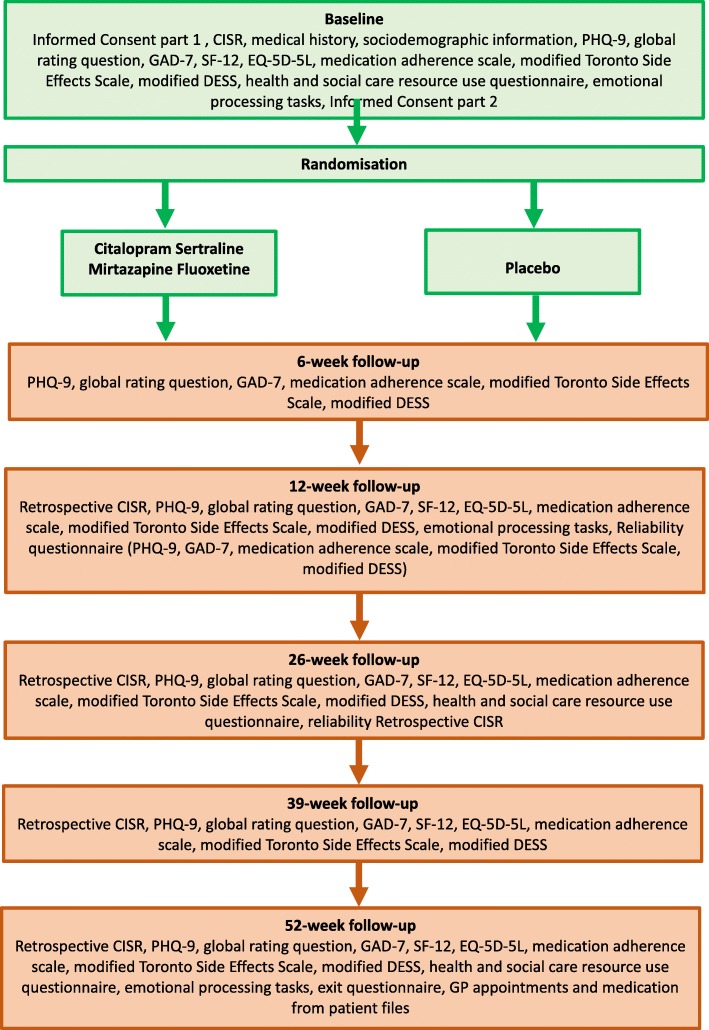
Summary of the baseline and follow-up schedule for the ANTLER trial. ANTLER ANTidepressants to prevent reLapse in dEpRession, CISR Clinical Interview Schedule—Revised, DESS Discontinuation-Emergent Signs and Symptoms, EQ-5D-5L EuroQol 5D-5L, GAD-7 Generalized Anxiety Disorder-7, GP general practitioner, PHQ-9 Patient Health Questionnaire-9, SF-12 Short Form-12

The follow-up schedule is summarised in a flowchart (Fig. 1).

We will examine the test–retest reliability of the PHQ-9, GAD-7, retrospective CIS-R, adverse effects, withdrawal symptoms (we have included a scale consisting of 15 items) and adherence questionnaires. The participants will be asked to repeat those questionnaires at one of the follow-up appointments.

At the end of the 52 week follow-up period or on withdrawal from the study, participants will be advised to see their GP to discuss their continued treatment (Table 1).

**Table 1:**
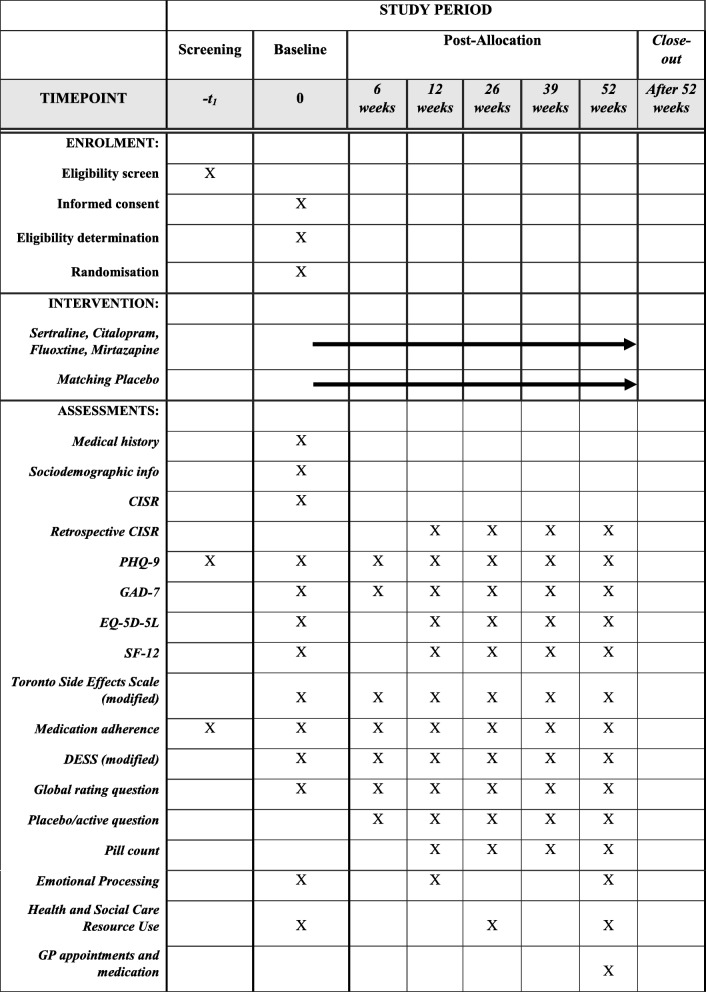
Full schedule of questionnaires used in the ANTLER trial

*ANTLER* ANTidepressants to prevent reLapse in dEpRession, *CISR* Clinical Interview Schedule—Revised, *DESS* Discontinuation-Emergent Signs and Symptoms, *EQ-5D-5L* EuroQol 5D-5L, *GAD-7* Generalized Anxiety Disorder-7, *GP* general practitioner, *PHQ-9* Patient Health Questionnaire-9, *SF-12* Short Form-12

### Mechanistic outcomes

We included three computerised emotional processing tasks (described in the following) to investigate the neuropsychological markers of antidepressant action. It has been consistently found [26, 28, 29] that antidepressants acutely affect performance even in healthy volunteers on emotion processing tasks, even though there is no subjective awareness of any change or improvement of mood. These markers of antidepressant response could be a factor that might be useful in predicting the likelihood of relapse.

The word recall task [26] tests memory of socially rewarding and socially critical information. The participant is presented with 20 likeable (e.g. cheerful, honest) and 20 dislikeable (e.g. untidy, hostile) personality characteristic words on a laptop screen in a random order for 500 ms. Words are matched according to length, usage frequency and meaningfulness, and they differ at each time point. After each word, participants indicate whether they would ‘like’ or ‘dislike’ to hear someone describing them in this way by pressing a key on the keyboard. At the end of the task, participants are asked to recall as many words as possible in 2 min. This is a surprise recall task (at baseline), to test incidental memory. The number of positive and negative words accurately recalled (hits) and the number of false responses (intrusions) are also recorded.

In the go–no-go task [25], each trial includes three events: the presentation of a fractal image; the presentation of a target; and a probabilistic outcome. At the beginning of each trial, one of four possible fractal images is presented on a computer screen, which indicates whether the best choice in a subsequent target detection task is a go (pressing a key on the keyboard) or a no-go (withholding a response to the target). The fractal also indicates the valence of any outcome dependent on the participant’s behaviour (reward/no reward or punishment/no punishment). The meaning of fractal images (go to win, no-go to win, go to avoid punishment, no-go to avoid punishment) is randomised across participants, and participants have to learn these by trial and error. Participants are informed that the correct choice for each fractal image is either a go (button press) or a no-go (withhold button press). Actions are required in response to a target circle that follows the fractal image. After a brief delay, the outcome is presented (an upward arrow indicates a win, a downward arrow indicates a loss and a horizontal bar indicates the absence of a win or a loss). On go-to-win trials, a button press is rewarded; on go to avoid punishment, a button press avoids punishment; in no-go to win, withholding a button press is rewarded; and in no-go to avoid losing trials, withholding a button press avoids punishment. The task consists of 240 trials in total (60 trial per condition). The participant can win between £1 and £10.

For the face task, prototypical ‘happy’ and ‘sad’ composite images were generated from 20 individual male faces showing a happy facial expression and the same individuals showing a sad expression from the Karolinska Directed Emotional Faces [30], using established techniques [31]. These were used as end-points of a linear morph sequence that changed in displayed emotion incrementally from unambiguously ‘happy’, through ambiguity, to unambiguously ‘sad’. The task has 15 images and each image is presented three times, resulting in 45 trials in total. Each stimulus is presented for 500 ms and followed by a pattern mask (250 ms) to disrupt any visual after images. Participants are required to judge faces from a morphed sequence as either sad or happy.

The results of the mechanistic outcome will not be presented in the main trial paper that will describe the primary and secondary outcomes, because these analyses do not address the primary aim of the trial, which is the clinical effectiveness and cost-effectiveness of long-term maintenance antidepressants treatment compared with a placebo. The mechanistic outcomes will be published in a separate paper or papers after the main trial results have been published. The paper(s) will aim to investigate hypotheses concerning the mechanism of action of antidepressant medication.

### Withdrawal of trial participants

Participants can withdraw from the trial at any time for any reason, without their medical care being affected. Where possible, data already collected will continue to be used in the trial and participants who stop taking the trial medication will be asked if they are still willing to meet with the researcher and provide follow-up data. Once participants have stopped their trial medication, they may not resume trial treatment. If participants withdraw, the reason for and type of withdrawal will be documented.

If the PI is concerned about the clinical condition of a patient such that they should not be on a placebo, we will withdraw that patient from the trial and will advise them to see their GP to receive appropriate treatment outside of the trial. The decision to withdraw will be based on factors such as depressive symptoms and suicidality and any other factor which the GP or PI thinks makes withdrawal in the best interests of the patient.

### Packaging, labelling and dispensing

The labelling of medication packs will be Medicines and Healthcare Products Regulatory Authority (MHRA) approved and will conform to Annex 13 of Good Manufacturing Practice (GMP) standards and Article 13.3 of Directive 200/20/EC [32]. Each medication pack will have a Medicine ID number, randomly generated to ensure active and placebo medicine packs are indistinguishable. This random number will link the pack label and tear-off portion containing the unblinded contents information. The tear-off label will be removed by the dispensing pharmacy at the point of dispensing.

The manufacturer will ship labelled and numbered packages to the dispensing pharmacy where the trial medication will be stored under controlled conditions. The pharmacy will dispense individual patient packs and oversee the packaging and posting of those packs. After randomisation, the participant will receive a pack containing 8 weeks’ supply of the trial medication. The trial medication will be posted every 2 months.

Full IMP accountability records will be maintained at the dispensing pharmacy: receipt, dispensing, distribution, return and destruction records. The receipt of the trial medication by the participant will be logged by the research team.

### Concomitant medication

The participants will already have been taking the antidepressant medication for at least 9 months before entering the trial. It is possible that some participants might be taking medication before entry to the study, which may have interactions or cautions with their antidepressant. If this does occur, the PI will make a clinical judgement about whether that person should be entered into the study. We will also notify the participant’s GP in writing of such cautions or possible interactions.

The only strict contraindication for the antidepressants used in the study is for monoamine oxidase inhibitors, so use of these is excluded.

### Adverse events

All adverse events (AEs) (untoward medical occurrence in a participant, which does not necessarily have a causal relationship with the treatment) of special interest will be recorded on a structured AE assessment (i.e. a list of physical symptoms) that is included in every follow-up assessment. If a participant consults their GP with a known AE, this will be recorded in the medical notes only but not communicated to the PI.

As this trial is a phase IV trial of licensed medications used within their licensed indication with a well-established safety profile, AEs will not be recorded in the CRF apart from those AEs of special interest included in the follow-up assessments.

All serious adverse events (SAEs) will be recorded by researchers on the Sponsor SAE reporting form and reported to the Sponsor within 24 h of their knowledge of the event. The CI and trial manager will also be informed. The CI/PI may contact the patient’s GP, depending upon the nature of the SAE, to obtain more information regarding the event. The Sponsor will notify the Research Ethics Committee (REC) and MHRA of all suspected unexpected serious adverse reactions (SUSARs). SUSARs that are fatal or life-threatening must be notified to the MHRA and REC within 7 days after the Sponsor has learned of them. Other SUSARs must be reported to the REC and MHRA within 15 days after the Sponsor has learned of them.

### Trial stopping rules

The trial may be prematurely discontinued by the Sponsor, Chief Investigator, Regulatory Authority or Funder on the basis of new safety information or for other reasons given by the Data Monitoring Committee and/or Trial Steering Committee regulatory authority or ethics committee concerned.

The trial may also be prematurely discontinued due to a lack of recruitment or upon advice from the Trial Steering Committee, who will advise on whether to continue or discontinue the trial and make a recommendation to the Sponsor. If the trial is prematurely discontinued, active participants will be informed and no further participant data will be collected.

### Statistical analysis

We will follow CONSORT guidelines in analysing the data and reporting the trial findings (http://www.consort-statement.org/). We will also prepare a CONSORT flow diagram. This will include the number of patients randomised to each arm of the trial, and the numbers who have follow-up data available. Initial analyses will look at summary statistics for all variables, both overall and by randomised group. Summary statistics for continuous variables will be the mean, median, SD, lower quartile and upper quartile, and will be reported appropriately according to distribution.

The primary outcome will be the time in weeks to the beginning of the first episode of depression after randomisation. The primary outcome will be assessed using a modified and shortened standardised psychiatric assessment (CIS-R) that will ask the participants retrospectively over the previous 3 months and will be used at follow-up points of 12, 26, 39 and 52 weeks. The retrospective CIS-R is based on five out of 14 CIS-R sections (depression, depressive ideas, concentration, sleep and fatigue), but asked retrospectively about symptoms at the worst point in the previous 3 months. The assessment also asks participants about the time before the assessment when the symptoms began and this is used to determine the time to relapse. This shortened CIS-R includes questions asking about depressive symptoms, such as restlessness, suicidal thoughts, hopelessness, feeling low for prolonged periods, unresponsiveness of mood, retardation, loss of sexual interest, lack of concentration, reduced self-esteem and feeling of guilt. An episode will be defined as those having two or more depressive symptoms included in the retrospective CIS-R for a period of at least 2 weeks. The precise definition will be included in the analysis plan.

Frank et al. [33] provided a theoretical conceptualisation and rationale for definitions of the five stages (response, remission, recovery, relapse and recurrence) in the course of depressive illness. However, in practice it is challenging to distinguish between relapse and recurrence because assessments rely on retrospective recall and symptoms usually vary over time. We will therefore not differentiate whether the first episode of depression after randomisation is a relapse (a return of symptoms of an ongoing, although suppressed, episode) or recurrence (a new episode of depressive disorder).

We propose to analyse the primary outcome using an exact Cox proportional hazards model (to account for ties), adjusting for the depressive symptom score from the CIS-R at baseline. We will undertake further supportive analyses including the minimisation variables as fixed patient-level explanatory variables.

Baseline predictors of missingness of the primary outcome will be examined.

The secondary outcomes will be the following:

- Depressive symptoms (PHQ-9) [19].
- Anxiety symptoms (GAD-7) [21].
- EQ-5D-5L for quality-adjusted life years (QALYs) [22].
- Adverse effects: physical symptoms (thought to be adverse effects of antidepressants) [23]. For each symptom we will ask the participant about the presence and frequency of the physical symptom and also whether they attribute the symptom to the medication.
- Health-related quality of life (SF-12) [34].
- Withdrawal symptoms based on the DESS (15 items) [24].
- Healthcare resource use collected from GP electronic records and directly from participants.
- Client Service Receipt Inventory (modified) for healthcare and other resource use.

Secondary outcomes will be analysed for each follow-up point using mixed-effects linear regression in which the baseline value and follow-up value will both be outcomes. The interaction between time and group will be estimated. Variables indicating time and the randomised group will be included in the models as fixed effects. We will conduct a supportive analysis using all observations for a participant in a further mixed-effects regression model for each outcome.

A detailed statistical and health economic analysis plan will be written and signed off by the IDMC for the trial before the database is locked. The plan will be logged in the UCL repository and/or on the ANTLER website.

### Economic evaluation

We will calculate the mean incremental cost per quality-adjusted life year (QALY) gained of antidepressant maintenance compared with placebo over 12 months from an NHS and social care perspective using trial data.

Healthcare resource use will be collected from GP electronic records and a modified version of the Client Service Receipt Inventory (CSRI), and will include information on primary and acute care health service contacts, pharmaceutical prescriptions, mental health community and inpatient service use, social care, employment and welfare payments. Services will be costed using nationally published sources. The cost of antidepressant maintenance will be calculated for the treatment group. For the primary analysis, costs will be from the NHS and social care perspective. A secondary analysis from the societal cost perspective will also be conducted.

QALYs will be calculated as the area under the curve using utility scores calculated from the EQ-5D-5L [22] collected at each time point, adjusting for baseline values.

Incremental costs and QALYs will be calculated using ordinary least squares (OLS) regression and adjusting for baseline depressive symptom score, baseline costs for the cost analysis and utility scores for the QALYs.

We will conduct one and two-way sensitivity analyses for any assumptions made and sub-group analyses as identified. Missing data will be handled in the same way as for the statistical analysis, with the primary analysis being an intention-to-treat analysis and supportive analyses taking into account assumptions about missingness. Bootstrapping will be used to construct confidence intervals and a cost-effectiveness acceptability curve of the probability that antidepressant maintenance is cost-effective for a range of values of willingness to pay for a QALY gained.

### Justification of sample size

A systematic review by Geddes et al. [10] estimated in an active group compared with placebo a reduction in odds of relapse of 70%, Kaymaz et al. [9] 65%, Glue et al. [11] 65% and NICE [8] 50%. Between 15 and 22% of those on active drug relapsed in 12 months. To detect the difference between relapse rates of 15% (continuation arm) and 30% (withdrawal arm) (hazard ratio 0.46), or 20% (continuation) and 35% (withdrawal) (hazard ratio 0.52), will require sample sizes of respectively 333 and 383 for 90% power at the 5% significance level. Allowing for 20% attrition, we therefore propose to recruit 479 participants [35].

### Data handling and quality assurance

The trial Sponsor is University College London and takes primary responsibility for ensuring that the design of the study meets appropriate standards and that arrangements are in place to ensure appropriate conduct and reporting. A monitoring plan has been agreed with the Sponsor. Local PIs will be responsible for the data quality at their centre. The trial will be run in accordance with Good Clinical Practice (GCP) and current regulatory guidance. All data will be handled according to the General Data Protection Regulation (GDPR) 2018 as well as UCL Information Security Policy and Trust Information Governance Policy. The investigators have full access to all of the data and are under no restrictions in their use of the data within the constraints of the relevant legal framework. We are open to approaches from bona fide researchers to have access to the data where this is consistent with our ethics and regulatory approvals and the legal framework.

### Publication policy

An ANTLER publication policy will be developed and agreed by co-applicants. The funder will be informed of the publications before they are submitted to journals. Publications will conform to the International Committee of Medical Journal Editors (ICMJE) guidelines for reporting and authorship.

### Ethics and regulatory approvals and reporting

The trial is being conducted in compliance with all applicable regulatory requirements. The Sponsor has ensured that the trial protocol, patient information sheet, consent form, GP letters and other documents have been approved by the appropriate regulatory body (MHRA in the UK) and a main research ethics committee, prior to any patient recruitment. The protocol and all agreed substantial protocol amendments have been documented and submitted for ethical and regulatory approval prior to implementation. Ethical approval was obtained from the National Research Ethics Service committee, East of England—Cambridge South (ref.: 16/EE/0032). Clinical trial authorisation was given by MHRA. The trial Sponsor is University College London. The trial has been registered: EudraCT Number 2015–004210-26; Protocol Number 14/0647 (version 6.0); Controlled Trials ISRCTN Registry, ISRCTN15969819.

It is the responsibility of the Chief Investigator/Principal Investigator or designee at each site to ensure that all subsequent amendments gain the necessary approval. This does not affect the individual clinician’s responsibility to take immediate action if thought necessary to protect the health and interest of individual patients.

The trial investigators and institutions will permit trial-related monitoring, audits, REC review and regulatory inspections, providing direct access to source data/documents. Trial participants are informed of this during the informed consent discussion.

Within 90 days after the end of the trial, the CI and Sponsor will ensure that the main REC and the MHRA are notified that the trial has finished.

The CI will supply the Sponsor with a summary report of the clinical trial, which will then be submitted to the MHRA and main REC within 1 year after the end of the trial.

There is a Trial Steering Committee chaired by Prof. Allan House of Leeds Institute of Health Sciences. The other independent members are Dr Geoffrey Wong, Prof. Jonathan Bisson and Ms Lucy Carr. The Independent Data Monitoring Committee is chaired by Prof. Chris Dowrick of University of Liverpool and the other members are Dr Rafael Perera-Salazar and Prof. Mike Crawford.

### Insurance

University College London holds insurance against claims from participants for injury caused by their participation in the clinical trial*.*

## Discussion

To our knowledge, the ANTLER trial will be the first large trial with a long follow-up of 12 months to investigate the effectiveness of antidepressants as maintenance treatment for patients who are already on long-term maintenance. Prof. Dee Mangin and the team carried out a similar trial in New Zealand in 2008, looking at maintenance vs gradual withdrawal of antidepressants in prevention of depression recurrence in primary care in patients with unipolar depressive disorder. The trial’s medication was fluoxetine and included patients (*N* = 263) who had been on antidepressants for 1 year or longer. It was registered on the Australian and New Zealand Clinical Trail Registry: ACTRN12608000613303. The sample size was reduced after the Christchurch earthquakes in 2010–2011 when a decision to stop recruitment at the primary site (Christchurch) was made in consultation with the Data Safety Monitoring Board—all patients enrolled continued in the trial in Christchurch and other sites. The results have not yet been published. The ANTLER trial will provide UK data on a more representative sample of antidepressants. Utilising both trials’ data together would create an opportunity for an individual patient meta-analysis for greater precision of estimates.

The ANTLER trial looks at the four most commonly prescribed antidepressants in primary care and these are off patent and relatively inexpensive. Due to the pharmacological similarities, we believe the results of the trial will be applicable to all major classes of antidepressants.

The results of the trial will address an important aspect of current clinical practice, by providing a valid and generalisable estimate of the clinical effectiveness and cost-effectiveness of long-term maintenance treatment with antidepressants in UK primary care. The trial participants will be recruited from a wide range of primary care settings across the four study centres, based in urban and rural, affluent and deprived areas across the UK, maximising the external validity of the findings (Additional file 1).

## Trial status

The trial began recruiting participants in March 2017 and recruitment will be ongoing until the end of February 2019. At the time of writing (September 2018), 370 participants have been randomised into the study. It is expected that data collection will be completed by February 2020.

This article describes protocol version 6 dated 3 August 2017; when all initial approvals (REC, HRA and MHRA) were received, the protocol was version 2 dated 22 February 2016. Following a substantial amendment, the protocol was amended with changes in the pharmacy arrangements, changes in Sponsor representative and a 6-week follow-up added, and it became version 5 dated 18 November 2016. As a result, when recruitment started the trial was following protocol version 5; since then it has been amended once to version 6 dated 3 August 2017: PHQ-9 assessment was removed from the eligibility criteria and instead is being used more flexibly at the screening stage to identify potentially eligible patients.

## Additional file

Additional file 1: SPIRIT 2013 Checklist: Recommended items to address in a clinical trial protocol and related documents (DOC 116 kb)

## Abbreviations

AE: Adverse event
CI: Chief Investigator
CIS-R: Clinical Interview Schedule—Revised
CONSORT: Consolidated Standards of Reporting Trials
CRF: Case Report Form
EQ-5D-5L: EuroQol 5D-5L
GAD-7: Generalized Anxiety Disorder-7
GCP: Good Clinical Practice
GP: General practitioner
HRA: Health Research Authority
IMP: Investigational Medicinal Product
MHRA: Medicines and Healthcare Products Regulatory Agency
NIHR: National Institute for Health Research
PHQ-9: Patient Health Questionnaire-9
PI: Principal Investigator
QALY: Quality-adjusted life year
REC: Research Ethics Committee
SAE: Serious adverse event
SD: Standard difference
SF-12: Short Form-12
SSRI: Selective serotonin reuptake inhibitor
SUSAR: Suspected unexpected serious adverse reaction

## References

[1] WHO. Depression: key facts. 2018; Available from: http://www.who.int/news-room/fact-sheets/detail/depression

[2] Moore M, Yuen HM, Dunn N, Mullee MA, Maskell J, Kendrick T (2009). Explaining the rise in antidepressant prescribing: a descriptive study using the general practice research database. BMJ.

[3] McCrea RL, Sammon CJ, Nazareth I, Petersen I. Initiation and duration of selective serotonin reuptake inhibitor prescribing over time: UK cohort study. Br J Psychiatry. 2016. 10.1192/bjp.bp.115.166975 27539294

[4] Tallon D, Wiles N, Campbell J, Chew-Graham C, Dickens C, Macleod U, et al. Mirtazapine added to selective serotonin reuptake inhibitors for treatment-resistant depression in primary care (MIR trial): study protocol for a randomised controlled trial. Trials. 2016;17. 10.1186/s13063-016-1199-2 PMC5526304 26842107

[5] NHS Digital. National Statistics Prescription Cost Analysis, England—2016 [Internet]. NHS Digital. 2017. http://www.hscic.gov.uk/catalogue/PUB17274. Available from: http://www.content.digital.nhs.uk/catalogue/PUB23631

[6] OECD. Health at a glance 2013: OECD indicators: OECD Publ; 2013.

[7] Coupland C, Dhiman P, Morriss R, Arthur A, Barton G, Hippisley-Cox J (2011). Antidepressant use and risk of adverse outcomes in older people: population based cohort study. BMJ.

[8] NICE. NICE. Management of depression in primary and secondary care: Clinical Guidelines 23. 2010

[9] Kaymaz N, Van Os J, Loonen AJM, Nolen WA (2008). Evidence that patients with single versus recurrent depressive episodes are differentially sensitive to treatment discontinuation: a meta-analysis of placebo-controlled randomized trials. J Clin Psychiatry.

[10] Geddes JR, Carney SM, Davies C, Furukawa TA, Kupfer DJ, Frank E (2003). Relapse prevention with antidepressant drug treatment in depressive disorders: a systematic review. Lancet..

[11] Glue P, Donovan MR, Kolluri S, Emir B (2010). Meta-analysis of relapse prevention antidepressant trials in depressive disorders. Aust N Z J Psychiatry.

[12] Cook BL, Helms PM, Smith RE, Tsai M (1986). Unipolar depression in the elderly. Reoccurrence on discontinuation of tricyclic antidepressants. J Affect Disord.

[13] Bialos D, Giller E, Jatlow P, Docherty J, Harkness L (1982). Recurrence of depression after discontinuation of long-term amitriptyline treatment. Am J Psychiatry.

[14] Kupfer DJ, Frank E, Perel JM, Cornes C, Mallinger AG, Thase ME (1992). Five-year outcome for maintenance therapies in recurrent depression. Arch Gen Psychiatry.

[15] Goldacre B. Bad Pharma: how drug companies mislead doctors and harm patients. 2012.

[16] Gøtzsche PC. Deadly medicine and organized crime: Radcliffe; 2013.

[17] Scherer RW, Langenberg P, Von Elm E. Full publication of results initially presented in abstracts. Cochrane Database Syst Rev. 2007. 10.1002/14651858.MR000005.pub3 17443628

[18] Cipriani A, Furukawa TA, Salanti G, Chaimani A, Atkinson LZ, Ogawa Y (2018). Comparative efficacy and acceptability of 21 antidepressant drugs for the acute treatment of adults with major depressive disorder: a systematic review and network meta-analysis. Lancet.

[19] Kroenke K, Spitzer RL, Williams JBW (2001). The PHQ-9: validity of a brief depression severity measure. J Gen Intern Med.

[20] Lewis G, Pelosi AJ, Araya R, Dunn G (1992). Measuring psychiatric disorder in the community: a standardized assessment for use by lay interviewers. Psychol Med.

[21] Spitzer RL, Kroenke K, Williams JBW, Löwe B (2006). A brief measure for assessing generalized anxiety disorder: the GAD-7. Arch Intern Med.

[22] Herdman M, Gudex C, Lloyd A, Janssen M, Kind P, Parkin D (2011). Development and preliminary testing of the new five-level version of EQ-5D (EQ-5D-5L). Qual Life Res.

[23] Crawford AA, Lewis S, Nutt D, Peters TJ, Cowen P, O’Donovan MC (2014). Adverse effects from antidepressant treatment: randomised controlled trial of 601 depressed individuals. Psychopharmacology.

[24] Rosenbaum JF, Fava M, Hoog SL, Ascroft RC, Krebs WB (1998). Selective serotonin reuptake inhibitor discontinuation syndrome: a randomized clinical trial. BiolPsychiatry..

[25] Guitart-Masip M, Economides M, Huys QJM, Frank MJ, Chowdhury R, Duzel E (2014). Differential, but not opponent, effects of l-DOPA and citalopram on action learning with reward and punishment. Psychopharmacology.

[26] Harmer CJ, Goodwin GM, Cowen PJ. Why do antidepressants take so long to work? A cognitive neuropsychological model of antidepressant drug action. Br J Psychiatry. 2009:102–8. 10.1192/bjp.bp.108.051193 19648538

[27] Button K, Lewis G, Penton-Voak I, Munafò M (2013). Social anxiety is associated with general but not specific biases in emotion recognition. Psychiatry Res.

[28] Harmer CJ, Shelley NC, Cowen PJ, Goodwin GM (2004). Increased positive versus negative affective perception and memory in healthy volunteers following selective serotonin and norepinephrine reuptake inhibition. Am J Psychiatry.

[29] Harmer CJ, O’Sullivan U, Favaron E, Massey-Chase R, Ayres R, Reinecke A (2009). Effect of acute antidepressant administration on negative affective bias in depressed patients. Am J Psychiatry.

[30] Lundqvist D, Flykt A, Ohman A. The Karolinska Directed Emotional Faces (KDEF), CD ROM from Department of Clinical Neuroscience, Psychology section: Karolinska Institutet; 1998.

[31] Tiddeman B, Burt M, Perrett D (2001). Prototyping and transforming facial textures for perception research. IEEE Comput Graph Appl.

[32] European Commission Enterprise and Industry Directorate-General. The rules governing medicinal products in the European Union. Brussels; 2010.

[33] Frank E, Prien RF, Jarrett RB, Keller MB, Kupfer DJ, Lavori PW (1991). Conceptualization and rationale for consensus definitions of terms in major depressive disorder: remission, recovery, relapse, and recurrence. Arch Gen Psychiatry.

[34] Ware JE, Kosinski M, Keller SD (1996). A 12-item Short-Form Health Survey: construction of scales and preliminary tests of reliability and validity. Med Care.

[35] Shih JH (1995). Sample size calculation for complex clinical trials with survival endpoints. Control Clin Trials.

